# Influence of Substrate Bias Voltage on Structure and Properties of (AlCrMoNiTi)N Films

**DOI:** 10.3390/nano14242002

**Published:** 2024-12-13

**Authors:** Xue Gao, Bin Li, Yiman Zhao, Xunwang Shi, Yujie Chen, Bin Liao, Erzhou Ren

**Affiliations:** 1 School of Intelligent Manufacturing, Luoyang Institute of Science and Technology, Luoyang 471023, China; 200900201817@lit.edu.cn (X.G.); ymzhao@lit.edu.cn (Y.Z.); jyrez@lit.edu.cn (E.R.); 2 School of Mechanical Engineering, The University of Adelaide, Adelaide, SA 5005, Australia; yujie.chen@adelaide.edu.au; 3 Key Laboratory of Beam Technology of Ministry of Education, College of Nuclear Science and Technology, Beijing Normal University, Beijing 100875, China; liaobingz@bnu.edu.cn

**Keywords:** (AlCrMoNiTi)N HEAN films, nanocomposite structure, co-FCVA, bias voltage, performance

## Abstract

(AlCrMoNiTi)N high-entropy alloy nitride (HEAN) films were synthesized at various bias voltages using the co-filter cathodic vacuum arc (co-FCVA) deposition technique. This study systematically investigates the effect of bias voltage on the microstructure and performance of HEAN films. The results indicate that an increase in bias voltage enhances the energy of ions while concomitantly reducing the deposition rate. All synthesized (AlCrMoNiTi)N HEAN films demonstrated the composite structure composed of FCC phase and metallic Ni. The hardness of the (AlCrMoNiTi)N HEAN film synthesized at a bias voltage of −100 V attained a maximum value of 38.7 GPa. This high hardness is primarily attributed to the synergistic effects stemming from the formation of strong metal-nitrogen (Me-N) bonding formed between the target elements and the N element, the densification of the film structure, and the ion beam-assisted bombardment strengthening of the co-FCVA deposition technique. In addition, the corrosion current density of the film prepared at this bias voltage was measured at 4.9 × 10^−7^ A·cm^−2^, significantly lower than that of 304 stainless steel, indicating excellent corrosion resistance.

## 1. Introduction

As is well known, traditional alloys are typically composed of one or two primary elements. In order to break from the shackles of traditional alloy systems, Yeh et al. [1] and Cantor et al. [2] propose the novel notion of high entropy alloys (HEAs). Unlike traditional alloys, HEAs are composed of five to 13 elements, with each element’s atomic percentage (at.%) controlled within the range of 5 to 35% [3]. This distinctive design concept endows HEAs with four fundamental effects: high entropy effects, severe lattice distortion, sluggish diffusion, and cocktail effects, which collectively result in HEAs often exhibiting simple phase structures and superior properties [4]. HEA films are the extension of bulk HEAs in the field of coating. Compared to bulk HEAs, HEA films exhibit superior properties. For example, the hardness of the CoCrFeMnNi HEA films is 8.5 GPa, which is higher than that of bulk HEAs [5].

Recently, high-entropy alloy nitride (HEAN) films, which are based on HEA film research, have attracted widespread attention [3,6,7]. Due to their simple phase structure [6], excellent corrosion resistance (the corrosion current density can reach around 10^−7^ A⋅cm^−2^) [8], outstanding hardness (the hardness can reach ultra-hard levels (>20 GPa)) [9], thermal stability (the films can exhibit anticrystallization ability at 700 °C) [10] and electro-optical properties (the conductivity and light reflectivity of the film can respectively reach 62.1 S/cm and 42.8%) [11], HEAN films have enormous application potential as a protective film in various fields (such as aerospace, automotive manufacturing, cutting tools, and molds) [8,12]. According to previous research, differences in preparation techniques [11,13,14,15,16], such as magnetron sputtering (MS), vacuum arc deposition (VAC), and filtered cathodic vacuum arc (FCVA), as well as deposition parameters [15,17,18,19,20] (nitrogen (N_2_) gas flow rates, target power, bias voltage and deposition temperature), significantly affect the microstructure and performance of HEAN films.

Compared to MS and VAC, the co-filtered cathodic vacuum arc (co-FCVA) technique, which was optimized based on the FCVA technique, can effectively filter out neutral atoms and macro ions during the plasma transmission process, resulting in a high-quality plasma beam with a 100% ionization rate [21,22]. In particular, the energetic ion beam has a notable impact on the growth of films during the deposition process, which can enhance the densification of the films. Additionally, the target material used in the FCVA deposition technique can be freely selected, allowing for controlled and adjustable film compositions, thus facilitating the preparation of HEAN coatings [16].

According to previous studies [18,23], an appropriate bias voltage can improve the densification of the film while simultaneously detracting loose grain atoms that adhere to the substrate. However, the phenomenon of re-sputtering may occur as the bias voltage increases, which will weaken the densification of the films. Thus, the bias voltage during the film deposition process plays a significant role in determining the structure and performance of the film. Unfortunately, current studies on HEAN films primarily focus on the effects of compositional elements and nitrogen content on the microstructure and performance of the films, with limited reports addressing the influence of substrate bias voltage on the films’ microstructure and properties. Thus, the influence of bias voltage on (AlCrMoNiTi)N HEAN films microstructure and performance were systematically investigated in this study.

## 2. Experimental

### 2.1. Film Preparation

**Figure 1:**
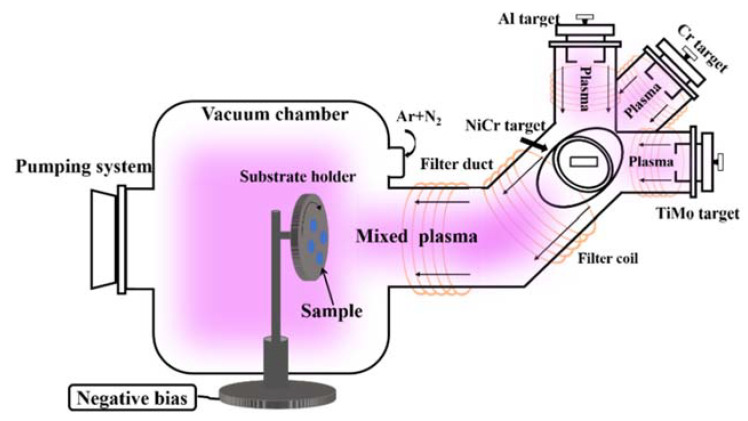
Schematic diagram of the co-FCVA equipment.

This study employed 304 stainless steel (304 SS) and silicon wafers as substrates for the preparation of AlCrMoNiTi HEAN films via co-FCVA deposition. Moreover, the HEAN films deposited on the surface of the silicon wafers were used to analyze the microstructure of the films, and the HEAN films deposited on the surface of the 304 SS were used to test the mechanical and corrosion properties of the films. The schematic diagram of the co-FCVA equipment is shown in Figure 1. Two alloy targets—TiMo (comprising 50 atomic percent (at.%) of Ti and 50 at.% of Mo) and NiCr (containing 80 at.% of Ni and 20 at.% of Cr)—along with two pure metal targets (Cr and Al), were selected for the deposition process. The purity of all the metal targets was 99.80%. The reaction gases Argon (Ar) and N_2_ were utilized at purities of 99.999%. Before deposition, the substrates underwent ultrasonic cleaning with acetone, alcohol and distilled water, followed by drying with N_2_ gas. In addition, before the actual film deposition, the bias voltages of −800, −600, −400 and −200 V were sequentially set to etch the substrate. The total etching time was 240 s. The flow rates of Ar and N_2_ were maintained at 60 and 120 standard cubic centimeters per minute (sccm), respectively, with a working pressure of 0.27 Pascals (Pa). The bias voltage for the HEAN films was varied from 0 V to −300 V. Detailed descriptions of the co-FCVA deposition system can be found in the referenced literature [16,24]. A comprehensive list of the deposition parameters for the (AlCrMoNiTi)N HEAN films is presented in Table 1.

### 2.2. Structure Characterization

A field emission scanning electron microscopy (FESEM, S-4800, Hitachi High-Tech Corporation, Tokyo, Japan) equipped with energy dispersive spectroscopy (EDS, EX-350, Hitachi High-Tech Corporation, Tokyo, Japan) was utilized to test the thickness, deposition rate, and morphology of the films deposited on the surface of the silicon wafers. The HEAN films deposited on the silicon substrate were broken by external force using a diamond knife to obtain a fresh cross-section, and then the dissociated sample is fixed on the sample holder with conductive tape for structure characterization. Furthermore, the post-nano indenter tests the morphology of the films fabricated on the surface of the 304 SS was also characterized using FESEM. The roughness and compositions of the films deposited on the surface of the silicon wafers was assessed by atomic force microscopy (AFM, Tosca™ 400, Anton Paar, Graz, Austria) and electron probe X-ray microanalyzer (EPMA, JXA8200, JEOL, Tokyo, Japan), respectively. SmartLab S2 grazing incidence x-ray diffractometer (GIXRD, Worcestershire, UK) was employed to analyze the phase structure of the films deposited on the surface of the silicon wafers. The scanning angle during the test process ranged from 20° to 90°. Moreover, the glancing incident angle and step size were 1° and 0.02°, respectively. Additionally, the grain sizes (D) of the films were calculated using the Scherrer equation [25]:

$$D=\frac{\alpha \times \lambda }{\varepsilon \times \cos\theta }$$

where *α* is the Scherrer constant (0.9), λ is the wavelength of the X-ray (1.5406 nm), θ is the peak position in radians and *ε* is the full width at half maximum.

### 2.3. Mechanical Properties

The mechanical properties (hardness (H), Young’s modulus (E), H/E, H^3^/E^2^ and elastic recovery (Re)) of the films prepared on the surface of the 304 SS were assessed using a nano-indenter (G200, Keysight Technologies, KLA Inc., Milpitas, CA, USA). To minimize substrate effects, during the tests, the indentation depth was controlled to less than 1/10 of the film thickness. In order to reduce errors, each test was repeated six times.

### 2.4. Corrosion Resistance

The corrosion behavior of the (AlCrMoNiTi)N HEAN films deposited on the surface of the 304 SS was investigated by using an electrochemical workstation (PARSTAT Model 2273, AMETEK, Berwyn, PA, USA). In addition, the tests were carried out under room temperature conditions. The setup included a platinum auxiliary electrode, the deposited films as the working electrode, and a saturated calomel electrode (SCE) as the reference electrode. The test was conducted in 5 wt.% H_2_SO_4_ solution, and the scanning rate was 1.0 mV/s. Additionally, Tafel extrapolation of the deposited films was used to determine the anodic and cathodic Tafel slopes (β_a_ and β_c_), corrosion current density (i_corr_), and corrosion potential (E_corr_). In addition, the polarization resistance (R_p_) of the films was calculated by Stern-Geary equation:

$$R_{p}=\frac{\beta_{a}\times \beta_{c}}{2.303\times (\beta_{c}+\beta_{a})\times i_{corr}}$$

## 3. Results and Discussion

The elemental concentration of the (AlCrMoNiTi)N HEAN films deposited under different substrate biases are listed in Table 2. It was observed that the contents of the film elements change only slightly with increasing substrate bias, indicating that the element contents are largely independent of the substrate bias. Previous studies have shown that the metal Ni element, classified as a non-nitride element, does not bond with nitrogen [26,27]. In contrast, the other target elements (Al, Cr, Mo and Ti) are strong nitride-forming elements that can bond with nitrogen to form Me-N compounds [6,12]. Therefore, based on Table 2, it can be inferred that the nitrogen content in all the deposited (AlCrMoNiTi)N HEAN films has reached a saturation state.

**Figure 2:**
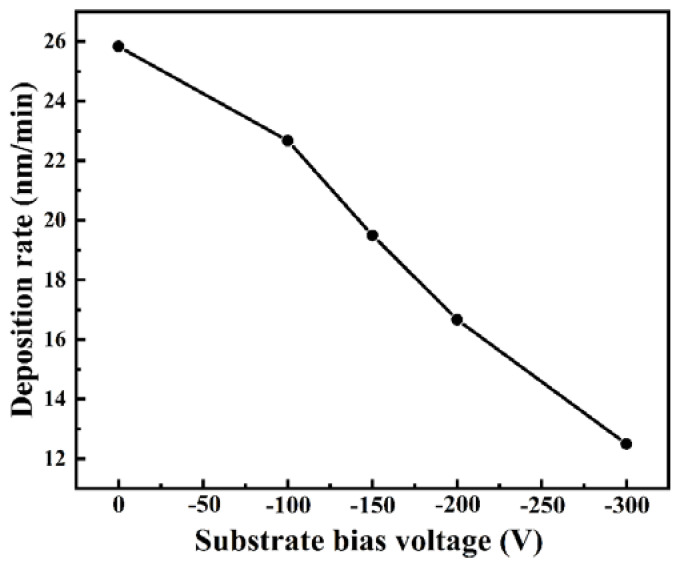
Deposition rate of the (AlCrMoNiTi)N HEAN films as a function of the bias voltage.

The deposition rates of the (AlCrMoNiTi)N HEAN films as a function of the bias voltage are illustrated in Figure 2. The deposition rates of these films gradually decrease from 25.83 to 12.50 nm/min as the negative bias voltage increases from 0 to 300 V. According to previous research, the re-sputtering of the films during the deposition process is correlated with the energy (U) of the ion bombardment on the growing film, which can be expressed as: U∝VD/P^1/2^ [17,28]. In this formula, D represents the power density of the target, V represents the bias voltage, and P represents the process pressure. During the deposition process, D and P remain constant. Thus, according to the formula, U is proportional to V. Consequently, the value of U increases with increasing V. This increase in U results in enhanced re-sputtering and adatom mobility, leading to a decrease in the deposition rate.

**Figure 3:**
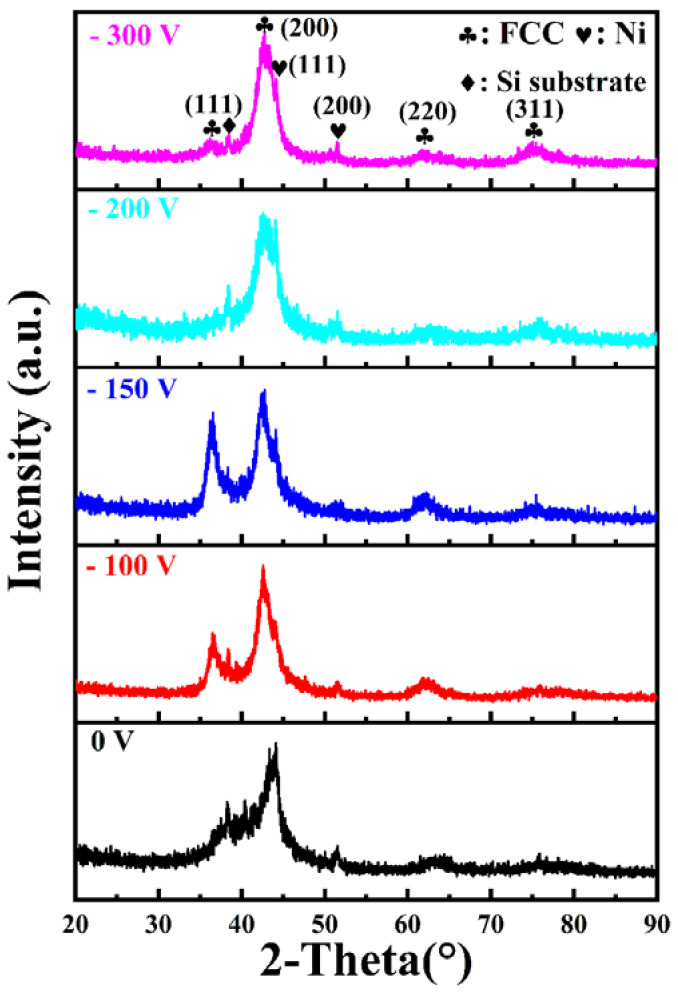
GIXRD patterns of (AlCrMoNiTi)N HEAN films prepared under various bias voltages.

GIXRD patterns of the (AlCrMoNiTi)N HEAN films prepared under various bias voltage are depicted in Figure 3. It is noteworthy that all deposited films exhibit a face-centered cubic (FCC) phase combined with a metallic Ni nanocomposite structure. According to a previous study, the peaks located at 44.5° and 51.9° correspond to the (111) and (200) planes of the metallic Ni phase, respectively [29]. The crystal structures of target elements and corresponding nitrides are summarized in Table 3. Notably, metallic Ni does not bond with nitrogen, so it exists in the deposited films as a metallic Ni phase. For the other target elements, AlN and MoN present hexagonal-close-packed (HCP) phase structures, while CrN and TiN show FCC phase structures, as summarized in Table 3 [30,31].

The deposited films exhibit a mixed phase of metallic Ni + FCC phase, indicating that the binary nitride FCC phase that effectively contains the other phase. The average grain sizes of the (AlCrMoNiTi)N HEAN films varied slightly, all within the range of ~10 nm (shown in Table 4). It is noteworthy that the average grain size of the (AlCrMoNiTi)N HEAN films fabricated by co-FCVA deposition technique in this study is smaller than that of films with the same composition deposited by RFMS (~16 nm) [26]. Furthermore, the mixed phase (metallic Ni + FCC phase) structure of the deposited films in this study differs from that of films with the same elements prepared by RFMS, which exhibit single FCC phase structure [26].

For the films prepared by co-FCVA deposition technique, during the deposition process, the neutral particles and large particles were filtered out through the magnetic filter tube, resulting in a high-quality 100% ionized positive ion beam at the outlet [21]. Consequently, the target positive ions at the outlet of the magnetic filter tube exhibit a high ionization rate. Therefore, Ni exists as a metallic phase in the film because it does not react with the nitrogen element. Moreover, the grain growth was suppressed by the periodic bombardment of high-energy ion beams during the deposition process, leading to a smaller grain size in the films.

**Figure 4:**
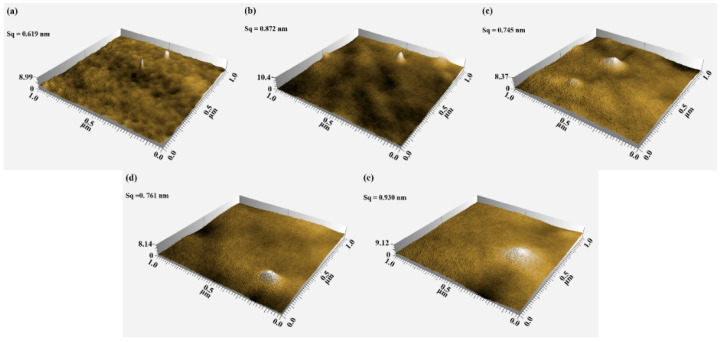
Surface morphology images of the (AlCrMoNiTi)N HEAN films obtained by AFM: (**a**) 0, (**b**) −100, (**c**) −150, (**d**) −200 and (**e**) −300 V.

The AFM surface morphology images of (AlCrMoNiTi)N HEAN films fabricated under various bias voltages are presented in Figure 4. These films exhibit low surface roughness, with Sq values in the nanometer range. Specifically, the Sq values for the (AlCrMoNiTi)N HEAN films fabricated at bias voltages of 0, −100, −150, −200 and −300 V were 0.619, 0.872, 0.745, 0.761 and 0.930 nm, respectively. Furthermore, the deposited films’ surfaces feature minimal particle presence, indicating superior coating quality.

**Figure 5:**
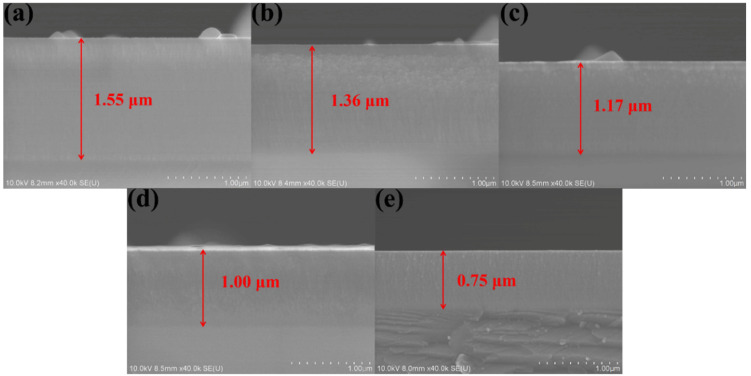
The cross-sectional FESEM images of the (AlCrMoNiTi)N HEAN films prepared at various bias voltage: (**a**) 0, (**b**) −100, (**c**) −150, (**d**) −200 and (**e**) −300 V.

The cross-sectional FESEM images of the (AlCrMoNiTi)N HEAN films fabricated under various bias voltages are illustrated in Figure 5. As the negative bias voltage increases from 0 to 300 V, the thickness of these nitride films decreases from 1.55 μm to 0.75 μm. Despite this decrease in thickness, the films show uniform and dense structures with no visible pores. Additionally, as the bias voltage increases, the FESEM images reveal an increase in the number of particles on the cross-sectional surface of the HEAN films. This phenomenon can be attributed to the increased energy of the ions and enhanced atom mobility with higher substrate bias voltages. According to previous studies, increasing the bias voltage appropriately can promote the densification and column refinement of the film structure, which is advantageous for the quality of the film. However, excessive substrate bias can lead to a decline in film quality, significantly impacting the films’ performance [23]. Despite the increased particle count with higher substrate bias, the overall structure of the films remains dense. Furthermore, all the deposited (AlCrMoNiTi)N HEAN films are firmly adhered to the substrates, with interfaces free of defects or cracks. According to a previous study, the film’s structure and the bonding strength between the film and the substrate are key elements affecting the corrosion resistance of the films [32].

**Figure 6:**
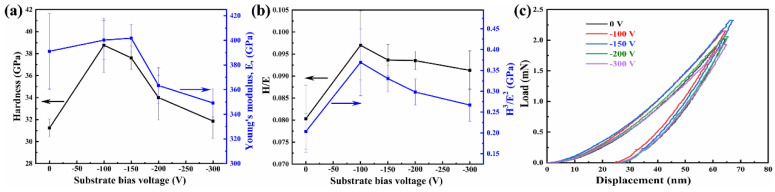
Mechanical properties of the (AlCrMoNiTi)N HEAN films fabricated at various bias voltages: (**a**) H and E, (**b**) H/E and H^3^/E^2^ and (**c**) load-displacement curves.

**Figure 7:**
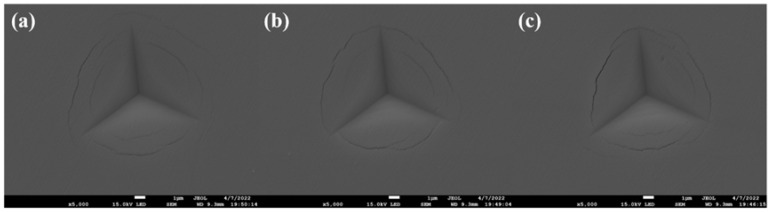
Nanoindentation FESEM images of the (AlCrMoNiTi)N HEAN films prepared under various bias voltages: (**a**) 0, (**b**) −100 and (**c**) −300 V.

The mechanical properties of the (AlCrMoNiTi)N HEAN films fabricated at various bias voltage are depicted in Figure 6. The nanoindentation FESEM images of the (AlCrMoNiTi)N HEAN films fabricated under various bias voltage are illustrated in Figure 7. Specifically, the elastic strain and resistance to plastic deformation of the films can be reflected by the ratios of H/E, H^3^/E^2^ and elastic recovery [33]. As seen in Figure 6, the values of H, E, H/E, H^3^/E^2^ and elastic recovery of the (AlCrMoNiTi)N HEAN films all exhibit a trend of first increasing and then decreasing as the negative bias voltage increases from 0 to 300 V. Notably, the values of H, H/E, H^3^/E^2^ and elastic recovery of the films prepared at the bias voltage of −100 V reach their maximum values of approximately 38.76 GPa, 0.96, 0.37 GPa and 61.95%, respectively. Moreover, the values of H, H/E, H^3^/E^2^ and elastic recovery decrease as further increasing the bias voltage. The H of the (AlCrMoNiTi)N HEAN films fabricated by co-FCVA deposition technique is higher than that of HEAN films with similar constituents synthesized by MS technique, which is around 15 GPa [26]. As shown in Figure 7, all nanoindentation morphologies of (AlCrMoNiTi)N HEAN films exhibit microcracks in the outer ring, while the crack lengths in the inner ring are similar. The appearance of the microcracks is due to the high hardness of the HEAN films.

Besides, the H values of the (AlCrMoNiTi)N HEAN films prepared in this study are also much higher than that of most HEAN films, including AlCrSiNbZr [13], TiZrHfNiCuCo [20], AlCrMnMoNiZr [34], FeCoNiCuVZrAl [35] and AlCrNbYZr [36] HEAN films, and similar to that of AlCrTiVZr [14], AlCrTiZrHf [37], TiVCrZrNbMoHfTaWAlSi [11], AlCrMoTaTiZr [38] and AlCrNbSiTiV [9] HEAN coatings. This indicates that the co-FCVA technique employed in this study effectively enhances the hardness of the (AlCrMoNiTi)N HEAN film, making it superior to many other HEAN films in terms of mechanical performance. It is well known that numerous factors influence the hardness of films, including elemental composition, film structure and morphology, deposition technique, and more [18,20,23,26]. Therefore, the high H of the (AlCrMoNiTi)N HEAN films prepared in this study can be attributed to the following factors:

(i) The HEAN films, composed of strong nitride-forming elements, typically exhibit high or even ultra-high hardness [15,23,39]. In this study, target elements Al, Cr, Mo and Ti are all strong nitride-forming elements [24], which can combine with N element to form strong Me-N bonds, thereby contributing to high hardness.

(ii) According to the Hall-Petch effect [40], the H value of the materials, within a certain range of grain sizes, increases with decreasing the grain size. When the grain size < 10 nm, the inverse Hall-Petch effect occurs [34]. Based on the GIXRD results, while the grain size of the fabricated HEAN films is all below 10 nm, it varies between 5 nm and 9 nm, indicating a relatively small change in grain size. Consequently, the microstructure of the film has a relatively minor impact on its hardness.

(iii) As reported, the densification of the film significantly impacts its H [23,41]. As depicted in Figure 4, the fabricated HEAN films exhibit a uniform and dense structure without any pores, leading to high hardness. However, the H of the (AlCrMoNiTi)N HEAN films prepared at high bias voltage decreases, which is attributed to a decrease in film quality under high substrate bias conditions.

(iv) Generally speaking, the microstructure and performance of films prepared by different deposition technologies vary [16,23,39,42,43]. In this paper, the (AlCrMoNiTi)N HEAN films were fabricated using the co-FCVA technique, which filters out neutral particles and large droplets, resulting in high-quality films [44]. In addition, ion beam-assisted bombardment with a certain energy level can inhibit grain growth, enhance film density and significantly improve film hardness during the film preparation process.

**Figure 8:**
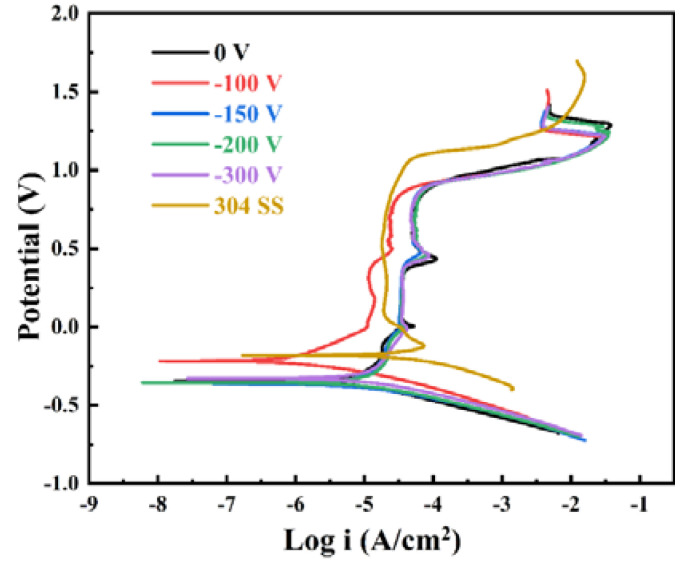
Potentiodynamic polarization curves of (AlCrMoNiTi)N HEAN films synthesized on the surface of 304 SS and 304 SS in 5 wt.% H_2_SO_4_ solution.

Figure 8 presents the dynamic polarization curves of (AlCrMoNiTi)N HEAN films fabricated on the surface of 304 SS and 304 SS in a 5 wt.% H_2_SO_4_ solution. Correlation to this, Table 5 displays the corrosion parameters of these materials. According to prior investigations, the corrosion performance of the film is conventionally evaluated through the i_corr_ parameter, which is proportional to the corrosion rate (R_p_) [45]. The value of R_p_ serves as an indicator of the film’s protective efficacy; a higher R_p_ corresponds to superior corrosion resistance [46]. The value of i_corr_ for the 304 SS substrate is 3.25 × 10^−5^ A·cm^−2^ (as discernible from Figure 8 and Table 5). For the synthesized (AlCrMoNiTi)N HEAN films, the trend of R_p_ values is closely aligned with that of the i_corr_ values. Specifically, the i_corr_ of the (AlCrMoNiTi)N HEAN film synthesized at the bias voltage of 0 V is measured at 3.0 × 10^−^⁶ A·cm^−2^. This value demonstrates a continuous decrease to 4.9 × 10^−7^ A·cm^−2^ when the bias voltage is set at −100 V, after which it exhibits an increase to 3.51 × 10^−^⁶ A·cm^−2^ at the bias voltage of −300 V. Noteworthy, the i_corr_ values for all the synthesized (AlCrMoNiTi)N HEAN films are significantly lower than that of 304 SS, indicating the outstanding corrosion performance of the (AlCrMoNiTi)N HEAN films. The corrosion property of the films is significantly influenced by microstructure, the interface bonding between the film and the substrate and the elemental constitute [34,47,48]. Based on the results of the GIXRD and FESEM experiments, all the synthesized (AlCrMoNiTi)N HEAN films in this study exhibit a simple FCC phase structure and are tightly adhered to the substrates. Therefore, the i_corr_ values of the films do not differ significantly. As mentioned earlier, appropriately increasing the substrate bias voltage can promote densification and column refinement of the films. However, excessive substrate bias voltage may lead to a decrease in film quality. Consequently, the i_corr_ values of the films synthesized at high bias voltage show a slight increase.

## 4. Conclusions

The (AlCrMoNiTi)N HEAN films were synthesized via co-FCVA under varying substrate bias voltages. The deposition rate decreased from 25.83 to 12.50 nm/min as the negative bias voltage increased from 0 to 300 V. All the (AlCrMoNiTi)N HEAN films exhibited a composite structure of metallic Ni nanoparticles and FCC phase structure. Although the number of particles increased with higher bias voltages, the films as a whole manifested a dense structure and adhered tightly to the substrates. The maximum H, H/E ratio, H^3^/E^2^ ratio and elastic recovery values of the films prepared at the bias voltage of −100 V reached 38.76 GPa, 0.37 GPa and 61.95%, respectively. Additionally, compared to the 304 SS substrate, the i_corr_ values of the (AlCrMoNiTi)N HEAN films were reduced by one order of magnitude.

## Figures and Tables

**Table 1:** Detailed parameters for the (AlCrMoNiTi)N HEAN films deposition.

Parameters	Film Deposition
Base pressure (Pa)	1.5 × 10^−3^
Working pressure (Pa)	0.27
TiMo/NiCr/Al/Cr target current (A)	140/80/100/100
N_2_/Ar gas flow (sccm)	120/60
Substrate bias (V)	0, −100, −150, −200, −300
Deposition time (min)	60

**Table 2:** The compositions (at.%) of the (AlCrMoNiTi)N HEAN films.

Substrate Bias (V)	Al	Cr	Mo	Ni	Ti	N
0	8.33	14.34	10.53	16.32	7.62	42.86
−100	8.68	13.9	11.37	14.71	7.75	43.59
−150	7.18	13.65	11.83	14.18	7.98	45.18
−200	5.53	14.15	13.22	16.35	8.38	42.37
−300	5.29	12.59	15.25	16.12	7.38	43.37

**Table 3:** Crystal structures of target elements and corresponding nitrides.

	Al	Cr	Mo	Ni	Ti	N
Radius (Å)	1.43	1.27	1.40	1.28	1.46	0.7
Structure	FCC	BCC	BCC	FCC	HCP	-
Nitride	AlN	CrN	MoN	-	TiN	-
Structure	HCP	FCC	HCP	-	FCC	-

**Table 4:** The crystalline size of resultant HEAN films.

Bias Voltage (V)	Crystalline Size (nm)						Average Crystalline Size (nm)
	FCC				Ni		
	(111)	(200)	(220)	(311)	(111)	(200)	
0	-	2.82	2.76	-	6.12	19.96	7.92
−100	5.42	5.10	3.83	1.46	7.82	14.46	6.35
−150	6.56	5.15	3.91	1.50	9.69	4.32	5.19
−200	5.67	4.71	4.13	1.45	16.76	13.70	7.74
−300	3.64	3.57	4.03	2.30	35.10	4.08	8.79

**Table 5:** Electrochemical corrosion properties of (AlCrMoNiTi)N HEAN films synthesized on the surface of 304 SS and 304 SS in 5 wt.% H_2_SO_4_ solution.

Sample	β_a_ (mV)	β_c_ (mV)	R_p_ (KΩ·cm^2^)	i_corr_ (10^−6^ A/cm^2^)	E_corr_ (V)
304 SS	126	36	0.38	32.50	−0.170
0 V	120.15	49.89	5.10	3.00	−0.350
−100 V	114.28	38.5	25.52	0.49	−0.222
−150 V	78.55	28.49	3.81	2.38	−0.369
−200 V	72.45	36.79	3.73	2.84	−0.360
−300 V	99.89	36.55	3.31	3.51	−0.335

## Data Availability

Data is contained within the article.
